# How can children benefit from total-body PET?

**DOI:** 10.1093/bjr/tqaf242

**Published:** 2025-09-25

**Authors:** Johannes H van Snick, Oleksandra V Ivashchenko, Joyce van Sluis, Limme B de Langen, Georgiana C van den Oever, Mostafa Roya, Andor W J M Glaudemans, Riemer H J A Slart

**Affiliations:** Department of Nuclear Medicine and Molecular Imaging, Medical Imaging Center, University Medical Center Groningen, University of Groningen, 9700 RB Groningen, The Netherlands; Department of Nuclear Medicine and Molecular Imaging, Medical Imaging Center, University Medical Center Groningen, University of Groningen, 9700 RB Groningen, The Netherlands; Department of Nuclear Medicine and Molecular Imaging, Medical Imaging Center, University Medical Center Groningen, University of Groningen, 9700 RB Groningen, The Netherlands; Department of Paediatrics, University Medical Center Groningen, University of Groningen, 9700 RB Groningen, The Netherlands; Department of Nuclear Medicine and Molecular Imaging, Medical Imaging Center, University Medical Center Groningen, University of Groningen, 9700 RB Groningen, The Netherlands; Department of Nuclear Medicine and Molecular Imaging, Medical Imaging Center, University Medical Center Groningen, University of Groningen, 9700 RB Groningen, The Netherlands; Department of Nuclear Medicine and Molecular Imaging, Medical Imaging Center, University Medical Center Groningen, University of Groningen, 9700 RB Groningen, The Netherlands; Department of Nuclear Medicine and Molecular Imaging, Medical Imaging Center, University Medical Center Groningen, University of Groningen, 9700 RB Groningen, The Netherlands; Biomedical Photonic Imaging Group, Faculty of Science and Technology, University of Twente, Enschede, The Netherlands

**Keywords:** paediatrics, LAFOV PET/CT, imaging, procedures, clinical applications

## Abstract

The introduction of long axial field-of-view (LAFOV) PET/CT scanners marks a major advancement in paediatric nuclear medicine. These systems provide greatly enhanced sensitivity, enabling superior image quality with reduced radiopharmaceutical doses and substantially shorter scan times. This is particularly advantageous in children, who are more radiosensitive and often struggle with prolonged procedures that may require sedation. LAFOV PET/CT allows whole-body imaging in a single bed position, reducing motion artefacts, improving patient comfort, and lessening procedural anxiety. Such benefits align with the ALARA (As Low As Reasonably Achievable) principle, critical for minimising radiation exposure in children given their increased sensitivity and longer life expectancy. Although associated with higher initial costs and increased data demands, LAFOV technology offers significant clinical advantages, including the potential for personalised imaging protocols tailored to each child’s needs. This review discusses the technical attributes of LAFOV PET/CT and its expanding role in paediatric imaging, addressing both opportunities and challenges. By overcoming previous limitations related to scan duration and radiation dose, LAFOV PET/CT is poised to transform paediatric diagnostics, enabling safer, faster and more comprehensive assessments.

## Introduction

Paediatric PET/CT imaging presents a distinct set of clinical, technical and ethical challenges that differ considerably from those encountered in adult practice. Children are not simply smaller versions of adults—their unique physiology, behavioural patterns and increased sensitivity to ionising radiation necessitate a bespoke and carefully considered approach to both imaging protocols and patient care.^1,2^

Traditionally, PET/CT has been underutilised in children, particularly outside oncology, due to logistical challenges, long scan times and concerns over cumulative radiation exposure.^3,4^ Moreover, young children often find it difficult to remain motionless during lengthy scans, compromising image quality and often necessitating sedation.^3–5^

Recent technological advances, notably long axial field-of-view (LAFOV) PET/CT scanners, are transforming paediatric nuclear medicine.^6,7^ These scanners offer markedly increased sensitivity, permitting high-quality imaging with reduced radiopharmaceutical doses, shorter acquisition times or both.^8^ This combination not only lowers radiation burden but also reduces motion artefacts and the need for sedation—substantially improving the safety, comfort and efficacy of imaging in children.^6^

LAFOV systems enable whole-body imaging in a single bed position, particularly advantageous in children, where reduced scan times, improved image alignment and lowered anxiety are essential. These innovations support the ALARA principle—maintaining radiation exposure As Low As Reasonably Achievable—vital due to children’s heightened radiosensitivity and longer life expectancy.^9^

Additionally, modern PET/CT offers flexibility to tailor imaging strategies^10^ Whether administering a rapid, slightly higher-dose scan for restless children or ultra-low-dose protocols for cooperative patients, LAFOV systems provide a versatile platform for personalised medicine.

The use of PET/CT has expanded significantly beyond its original role in oncology, now encompassing a wide range of diagnostic applications, including the detection of infectious and inflammatory foci in children presenting with fever of unknown origin.^11,12^ As its utility broadens, PET/CT is becoming an increasingly important modality in paediatric medicine.

This review examines technical advances and expanding clinical applications of LAFOV PET/CT in paediatric imaging, highlighting how it overcomes long-standing barriers to deliver safer, faster and more comprehensive diagnostics.

## Patient preparation

Undergoing a PET/CT scan can be a stressful experience for children and their families. The unfamiliar environment, intravenous injection, the need to remain still and intimidating equipment may induce anxiety or scan failure.^13,14^ High-quality imaging requires the child to remain motionless; therefore, thorough, age-appropriate preparation for the child and parents is essential.^4,15^

For example, a technologist or a physician assistant with specific expertise in PET-CT and skills in working with children can play a key role in ensuring that the entire procedure is conducted efficiently and with minimal stress.

Preparation begins with a pre-scan consultation, at least one day prior, explaining the procedure to the child and parents/caretakers and assessing the child’s ability to remain still.

A personalised scan protocol is devised accordingly: higher radiotracer doses and shorter scans for children unable to lie still, or lower doses with longer acquisitions for cooperative children.

Comfort and anxiety reduction strategies are integral, including parental presence during scanning, child-friendly lighting, favourite music and ceiling-projected films.^4,14^ Flexibility is maintained to adapt protocols based on the child’s condition on the scan day or even during scan acquisition.

Following these preparations, most paediatric PET/CT scans proceed without sedation. When needed, intranasal dexmedetomidine may aid relaxation.^16,17^ Careful preparations combined with rapid imaging by using the high sensitive LAFOV PET/CT transform paediatric PET/CT into a more accessible, efficient and child-friendly imaging procedure^6,18^

## Technical advantage of LAFOV PET/CT in paediatric imaging

Paediatric PET/CT imaging introduces unique challenges. As there is no standardised protocol, the dose of radiotracer and the acquisition time must be tailored to each child’s age, weight, clinical condition and ability to remain still. The available literature often provides inconsistent findings, underscoring the need for protocol optimisation specific to each system (see Table 1).^19–23^ Given these challenges, paediatric nuclear imaging requires a careful balance between diagnostic accuracy, minimising radiation exposure and keeping scan duration as short as possible.

**Table 1.** Recommended [^18^F]FDG PET/CT dose in children.

Source	PET/CT type	**Recommendations:** injected activity [MBq], effective scan speed [s/mm], total scan duration for a 70-cm region [min], post-injection delay [min]			
		5 kg	10 kg	20 kg	40kg
EANM dose card^19,20^	SAFOV^1^	59.5	76.2	100.6	137.1
		1.2-0.7 s/mm, 16.7-9.7 min, 60 min p.i.			
Cox, 2021^21^	SAFOV	18.2	34,5	65,3	123,5
		0,8 s/mm, 14.6 min, 60 min p.i.			
Tran-Gia, 2024^22^	SAFOV	18.5 MBq	37 MBq	74 MBq	148 MBq
		1.2-0.7 s/mm, 16.7-9.7 min, 60 min p.i.			
van der Kaap, 2024^48^	HS-LAFOV^2^	5.9	8.6	14.1	25.1
		0.3 s/mm, 5 min, 60 min p.i.			
Mingels, 2024^18^	TB^3^	2.5 MBq	5 MBq	10 MBq	20 MBq
		0.6 s/mm, 20 min, 120 min p.i.			
Van den Oever, 2025^4^^23^	UHS-LAFOV^5^	3.7 MBq	5 MBq	5 MBq	8.3 MBq
		0.05 s/mm 1 min	0.09 s/mm 1.7 min	0.2 s/mm 3.7 min	0.3 s/mm 5 min
		60 min p.i.			

1) SAFOV: short axial field-of-view PET.

2) HS-LAFOV: long axial FOV PET (Biograph Quadra) with a partial acceptance angle capability (i.e., high sensitivity mode).^66^

3) TB: total-body PET system (uEXPLORER, 194 cm FOV)^67^

4) Accepted abstract at EANM 2025.

5) UHS-LAFOV: with a full acceptance angle sensitivity (i.e., ultra-high sensitivity mode).

LAFOV PET/CT systems offer a solution to this problem.^24–26^ They combine very high sensitivity with the ability to scan the entire paediatric body in a single bed position, enabling either a substantial reduction in radiotracer dose or significantly shorter acquisition times (up to a maximum of a few minutes in total).^27–29^ Both strategies are highly valuable for young, uncooperative or critically ill children, as they improve comfort, reduce motion artefacts and enhance diagnostic quality. Additionally, continuous list-mode acquisition enables retrospective motion correction by allowing movement-affected frames to be excluded and reducing the need for repeat scans. Advanced reconstruction options broaden their utility; for example, extended photon acceptance angle modes allow high-quality imaging at lower doses.^30^

This enhances patient comfort, improves departmental throughput and adheres to ALARA principles^9^ LAFOV systems further demonstrate the clinical potential of this technology.

As clinical evidence continues to grow, LAFOV PET/CT is establishing itself as the new standard in paediatric nuclear medicine.^31^

In contrast, short axial field-of-view (SAFOV) PET/CT systems usually only cover 15–25 cm. This requires multiple bed positions and longer scan times. This can impact workflow efficiency and the patient experience.

As clinical evidence continues to grow, LAFOV PET/CT is establishing itself as the new standard in paediatric nuclear medicine—combining lower radiation exposure, greater comfort and high diagnostic confidence for some of the most vulnerable patient population.

## Radiation dose considerations

Ultra-low-dose PET/CT protocols in paediatric nuclear medicine are guided by the ALARA principle, which emphasises minimising radiation exposure wherever possible. Although the cancer risk from doses below 50 mSv remains debated, the International Commission on Radiological Protection (ICRP) supports the linear no-threshold (LNT) model, which assumes a linear relationship between dose and cancer risk with no safe threshold. This reinforces the necessity for dose optimisation, even at low exposure levels.^32^

Medical imaging is a significant source of man-made radiation, accounting for approximately 24% of the average annual dose in the Netherlands.^33^ This underscores the critical need to optimise imaging protocols, particularly for radiosensitive populations such as children. PET/CT imaging, especially with [^18^F]FDG and other radiotracers such as [^18^F]DOPA, plays a vital role in diagnosis, staging, treatment planning and follow-up but exposes children to ionising radiation from both the radiotracer and CT.^34^ Repeated scans can lead to substantial cumulative doses; currently, a single conventional paediatric PET/CT may deliver around 14.5 mSv, nearly twice that of an adult scan.^35–37^

Children are more vulnerable to radiation’s harmful effects due to their developing tissues and longer life expectancy, which increase the risk of late effects including secondary malignancies such as leukaemia and brain tumours.^35,38,39^ Survivors of paediatric cancer have consistently demonstrated higher rates of secondary neoplasms, particularly after extensive imaging regimes at a young age.^40–44^

**Figure 1.**
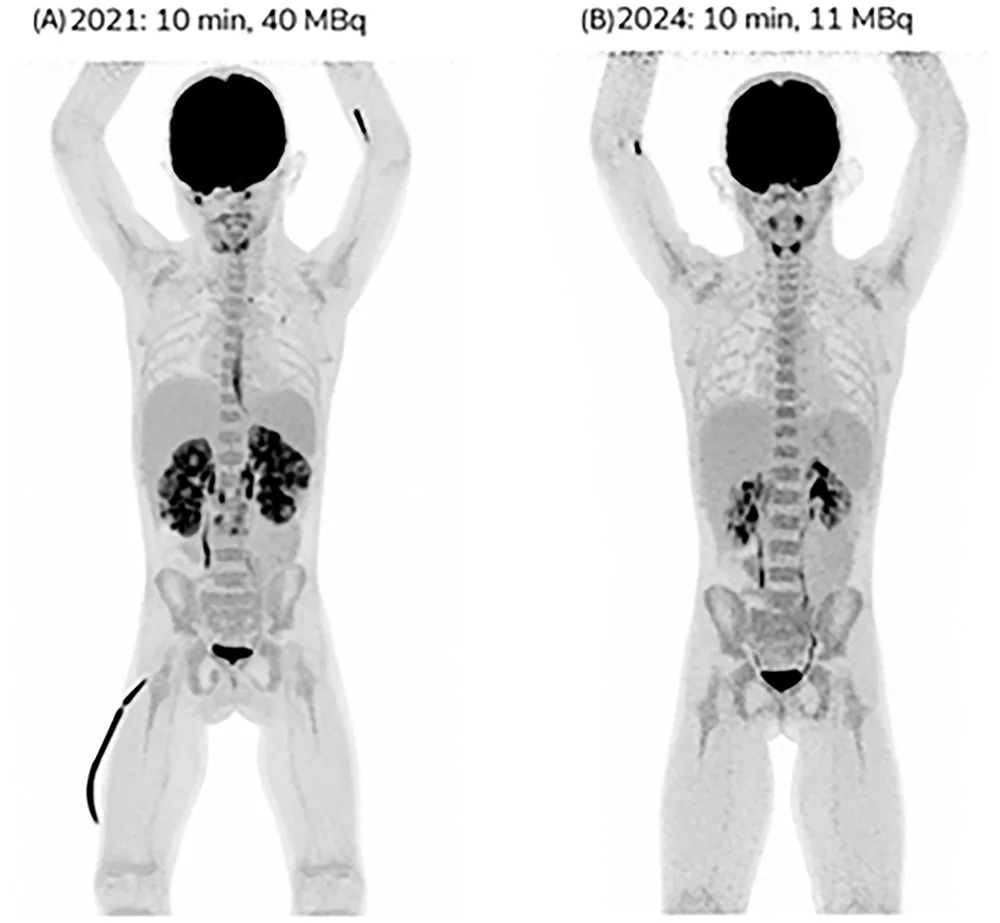
*Comparison of total-body PET/CT scans (Quadra) of the same patient taken three years apart. The first scan (A) used 40 MBq [^18^F]FDG and low-dose CT, resulting in a total effective dose of 3.08 mSv (PET 2.24 mSv, CT 0.84 mSv). The follow-up scan (B), after scanner optimisation, used 11 MBq [^18^F]FDG and optimised CT, reducing the total dose to 0.77 mSv (PET 0.62 mSv, CT 0.15 mSv). This represents a 75% overall dose reduction while maintaining diagnostic quality, consistent with ICRP 106 guidelines. Both scans addressed the same clinical question in a patient under 10 years old*.

The recent advances in PET technology support emerging proposals to revise paediatric radiotracer dosing. Recent analysis from the EuroNet PHL-C2 trial suggests a potential reduction in injected [^18^F]FDG activity for children, recommending a linear dose of 3.7 MBq/kg.^22^ This is nearly double the typical dose currently used in adults, which highlights the need for dose optimisation in paediatrics. LAFOV PET/CT scans can maintain clinical image quality at doses as low as 6.25–12.5% of standard reference doses in adults, with no significant loss in lesion detectability or tumour-to-background^29,45,46^ (see Figure 1). This translates into a potential reduction of administered radiotracer activity by approximately 93–98% relative to current EANM guidelines when utilising UHS mode on advanced systems such as the Biograph Vision Quadra.^27^

A recent national survey in the Netherlands evaluated current PET/CT acquisition and processing protocols, supporting the need for national or internation guidance for PET/CT protocol to be implemented with the best dose-image quality balance in mind.^30^ The study also indicated large coverage with digital PET/CT technology and modern scanner capacity, enabling dose reduction opportunities beyond the EANM Dose Card.

In a recent study by van der Kaap et al., the dose-reduction potential of LAFOV PET/CT for [^18^F]FDG imaging in children was preliminarily evaluated, demonstrating a possible reduction in administered activity of over 85% compared to the 2016 EANM Paediatric Dose Card.^47,48^

One should not forget that a PET/CT scan has two radiation components: internal from the injected PET tracer and external from the X-ray radiation delivered during a CT scan. With the dose reduction achieved by the total body/LAFOV PET technology development, CT’s radiation component in PET/CT becomes more and more prominent.^49,50^ Advanced X-ray filtering technology (i.e., tin filters) has been recently investigated to lower CT dose for attenuation correction purposes.

Several studies have reported reductions of up to 80–85% in AC-CT with the tin-filter technology in adults.^51^ However, such a level of dose reduction also results in significant degradation of image quality, which may compromise clinical use of the data.^52–54^

Additionally, novel approaches such as AI-based attenuation correction and CT-less methods using ^176^Lu transmission data are under exploration.^55–57^ While encouraging quantification accuracy has been demonstrated in adults, their feasibility and reliability in children remain to be established. These innovations represent a growing research area that may ultimately complement LAFOV PET/CT to minimise radiation dose while maintaining high diagnostic quality.

## Clinical applications and examples

### Oncology

Lymphoma is the third most common childhood cancer, comprising 10%–15% of all malignancies in children, surpassed only by leukaemia and central nervous system tumours.^58^ Many studies over the past decades have focused on risk-stratification and treatment-response adapted therapies, to minimise chemo- and radiation therapy overtreatment, to decrease adverse events and long-term sequela related to treatment.^45,46^ The use of diagnostic imaging has played a vital role in these efforts given that the stage of disease at presentation helps define the patient’s chemo- and radiation therapy regimen. The intensity of [^18^F]FDG uptake on PET correlates with the grade of malignancy and can help differentiate residual active disease from post-treatment change. Moreover, especially now with LAFOV PET/CT, a whole-body examination can be performed quickly with ultra-low radiation exposure. 

**Figure 2.**
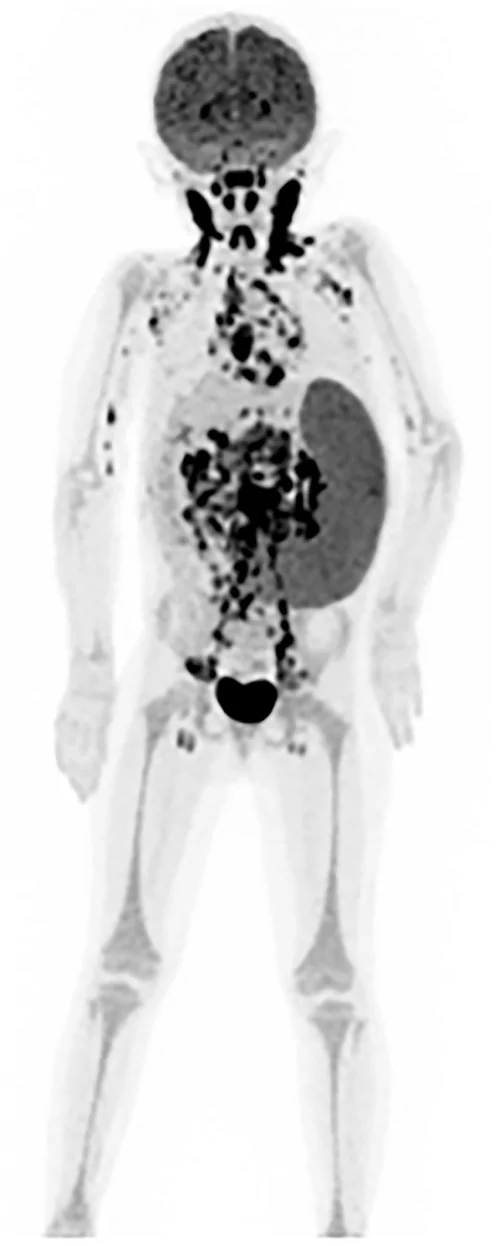
*Example of a 3-year-old girl (body weight 17 kilograms), post-orthotopic liver transplant, with pancytopenia and rising EBV titres, suspected of PTLD. Scanned on the Biograph Vision Quadra with just 18 MBq [^18^F]FDG over 5 minutes. Midazolam nasal spray (7.5 mg) was given 10 minutes before scanning. The scan was attended by her parents, who were there to support and comfort her, with her favourite music being played. Images show intensely metabolically active lymphadenopathy above and below the diaphragm, marked splenomegaly with diffuse FDG uptake, and likely reactive bone marrow activity*.

Another important indication for paediatric [^18^F]FDG PET/CT imaging is post-transplantation lymphoproliferative disorder (PTLD) (see Figure 2). PTLD is a major complication of immunosuppressive therapy after organ- or hematopoietic stem cell transplantation, and the most common post-transplant malignancy in children, with a higher reported incidence than in adults^59^ Early diagnosis of PTLD is challenging, yet essential for guiding treatment, management, and prognosis. While histological confirmation via biopsy is necessary, [^18^F]FDG PET/CT can support or rule out clinical suspicion of PTLD and help localise lesions suitable for biopsy. For evaluating treatment response, repeated assessment using fast, ultra-low-dose LAFOV [^18^F]FDG PET/CT provides an effective means for whole-body lesion monitoring.

### Cardiovascular

**Figure 3.**
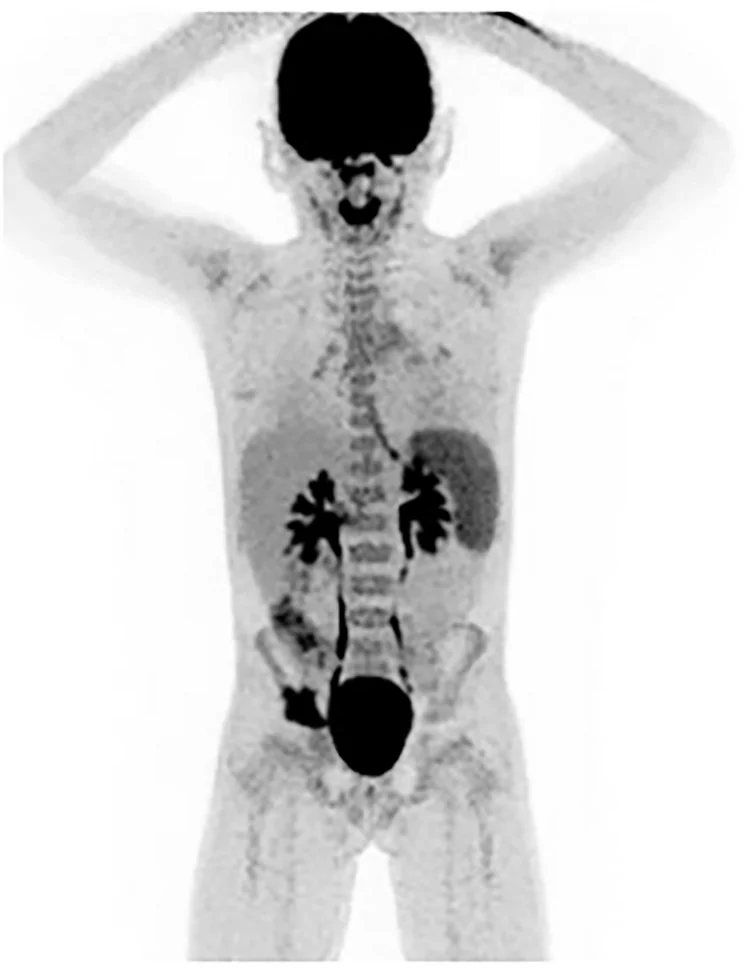
*[^18^F]FDG PET scan on the Biograph Vision Quadra PET/CT of an 8-year-old boy (body weight 27 kilogram) with suspected endocarditis (S. aureus, mitral valve vegetations). The injected dose was very low at just 12 MBq of [^18^F]FDG. As the patient was cooperative, no sedation was used. The patient was cooperative, so no sedation was used; distraction techniques included blue lighting and favourite music. Total scan time was 7 minutes. Findings: no endocarditis detected; intense uptake in the right acetabulum suggestive of osteomyelitis without CT substrate. Reactive lung hila and spleen*.

In paediatric cardiovascular imaging, LAFOV PET/CT application is emerging as a valuable tool, especially for evaluating complex congenital heart disease, cardiovascular inflammation (e.g., myocarditis, vasculitis), infections such as infectious endocarditis (see Figure 3), with the possibility to detect disseminated endocarditis across all body regions in a short acquisition time and 1-bed position, and finally assessing myocardial viability or (multi-organ) perfusion.^49,60,61^

In the context of heart disease, LAFOV PET/CT can detect systemic inflammatory or infectious processes that may affect the heart or arise due to cardiac conditions. Furthermore, it also supports decision-making in pre-surgical planning and post-operative monitoring. However, the need for paediatric-specific protocols, limited availability of tracers suited for paediatric cardiac imaging and possible higher costs are still challenges that remain.

Overall, LAFOV PET/CT shows significant promise in improving diagnostic accuracy, patient management and therapy monitoring in children with cardiovascular diseases, particularly when conventional imaging is inconclusive or insufficient.

### Infection & inflammation

**Figure 4.**
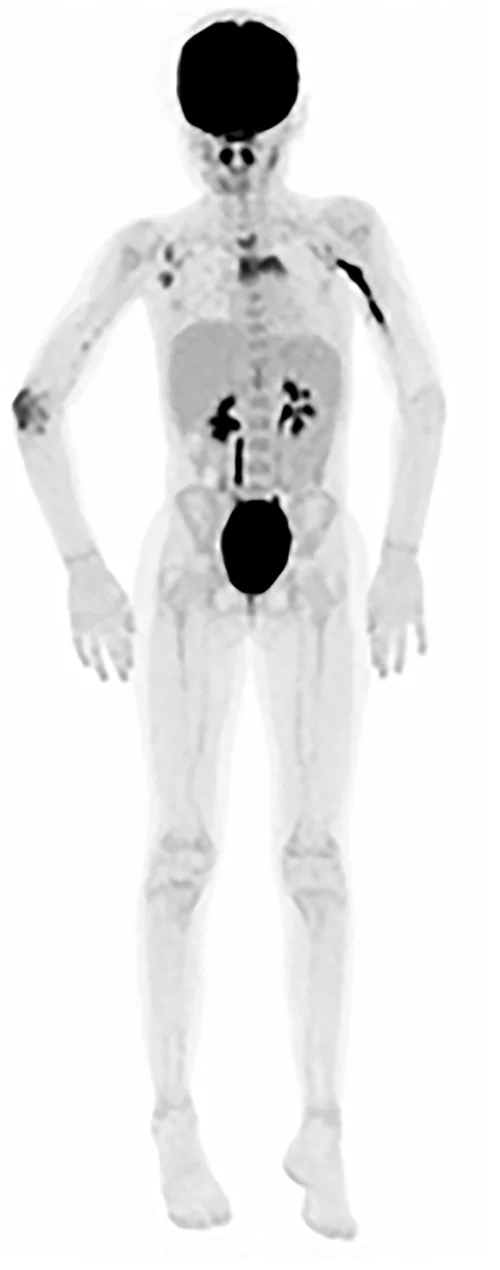
*A 9-year-old girl (body weight 26 kilogram). Clinical data: Persistent fever and infection parameters, septic arthritis of the right elbow. Transthoracic echocardiography showed no evidence of endocarditis. A PET/CT scan (Quadra) was performed in 12 minutes with only 15 MBq [^18^F]FDG and no use of any sedation. Her favourite music was played in the room to make the atmosphere more comfortable for her. Diagnosis: Uptake in the right elbow with known arthritis, no scatter marks or other affected joints. Reactive lymph nodes, right axillary and thymus. Injection artefact left upper arm*.

For infection and inflammation imaging in children, LAFOV PET/CT offers—besides the general benefits in reduction of radiation dose and reduced need for sedation—several important advantages compared to conventional scanners (see Figure 4). The increased sensitivity enables the detection of infections and inflammatory processes that were not detectable using conventional PET/CT scanners due to low metabolic activity of the disease process (chronic low-grade infections, infections with low bacterial load) or due to limited sensitivity (biofilms on prosthetic material, inflammation of cranial vessels, e.g., temporal and maxillary arteries.^62^ This can be crucial when a clinical diagnostic dilemma arises in the case of prolonged fever of unknown origin. For example, small vessel vasculitis or low-grade osteomyelitis are diagnosed more easily, when otherwise a bone marrow biopsy would have been conducted. Movement of the extremities is no longer an issue since only short-time acquisition is required for sufficient image quality. Due to ultrafast scanning, time outside the paediatric intensive care unit is highly reduced, and evaluation of critically ill children is now feasible. Whole-body parametric imaging opens up new possibilities for research.

## Discussion

LAFOV PET/CT represents a major advancement in paediatric nuclear medicine, addressing several of the unique challenges associated with imaging children. The use of ultra-low-dose [^18^F]FDG LAFOV PET/CT systems has been shown to provide diagnostically acceptable image quality even at substantially reduced radiotracer activities, without compromising diagnostic accuracy.^29^ This is particularly important in paediatric patients, who are highly radiosensitive and often undergo repeated imaging for follow-up, increasing cumulative radiation exposure. Recent studies suggest that the use of LAFOV PET/CT systems can achieve reductions in administered radiotracer activity of approximately 93–98% compared to current EANM paediatric guidelines^20,47,48^

Beyond dose optimisation, LAFOV PET/CT systems with extended axial coverage provide several practical advantages for paediatric imaging (see Table 2):

**Table 2.** Opportunities, challenges and solutions of LAFOV PET-CT in paediatric imaging.

Opportunities	Challenges	Solutions
Reduced scan duration minimises sedation need	Patient motion, especially in uncooperative or anxious children	Use child-friendly preparation, distraction techniques, motion correction software
Lower radiation dose due to increased sensitivity	Limited availability of LAFOV PET/CT in paediatric centres	Promote multicentre collaboration and funding to improve access
Whole-body imaging in a single bed position	Positioning challenges from small size and anatomical variability	Use tailored immobilisation devices and paediatric-specific positioning protocols
Enhanced metabolic and functional data	Complex interpretation due to developmental and age-related physiology	Develop age-specific reference databases and consult paediatric imaging specialists
Feasibility of dynamic or multi-tracer imaging	Motion artefacts despite faster acquisitions	Integrate real-time motion tracking and gating techniques
Shorter procedures reduce stress and improve departmental/examination workflow	High acquisition and maintenance costs	Demonstrate cost-effectiveness through improved throughput and reduced sedation needs
Enables safer longitudinal follow-up with reduced cumulative radiation exposure in chronic or oncologic indications	Lack of paediatric-specific normative data complicates quantitative analysis and standardisation	Establish paediatric imaging registries, encourage data sharing and demonstrate cost-effectiveness via improved throughput and reduced sedation

- **Whole-body imaging in a single scan**: Extended coverage allows for the simultaneous acquisition of the entire body in a single bed position, which is ideal for staging malignancies, detecting metastatic disease, and assessing systemic infections or inflammatory conditions commonly encountered in children.
- **Reduced scan duration**: The high sensitivity of LAFOV systems permits significantly shorter acquisition times. This is especially beneficial for younger or anxious patients, reducing the need for sedation or anaesthesia and enhancing patient throughput in clinical practice.
- **Flexible protocol design**: LAFOV technology supports tailored imaging protocols. Cooperative patients can benefit from ultra-low-dose imaging with longer scan times, while those requiring shorter scans can receive slightly higher doses to maintain image quality and reduce motion artefacts.

Despite these advantages, several challenges remain:

- **Lack of standardised international protocols**: Currently, there is no consensus on ultra-low-dose PET/CT protocols specific to LAFOV systems in paediatric populations. This results in variation between centres and underscores the need for multicentre collaboration to develop validated, harmonised protocols that ensure both safety and diagnostic efficacy.
- **Limited accessibility**: LAFOV PET/CT systems are currently available in a limited number of centres due to high acquisition and operational costs. Further research is needed to determine how similar dose reductions might be achieved on more widely available SAFOV systems with ultra-high sensitivity capabilities.
- **Heterogeneity of the paediatric population**: The wide variation in age, size and developmental stage among paediatric patients necessitates flexible, individualised imaging strategies. Future work should focus on refining personalised protocols to optimise both diagnostic quality and patient safety.
- **Child-friendly and well-organised imaging environment:** This includes a dedicated staff member for paediatric imaging, a specialised team and clear communication with both the child and their parents. Educating referring paediatricians about radiation risks and the value of PET/CT scans supports fully informed decision-making. Small steps—such as explaining the procedure, letting the child choose a movie and providing a familiar face—can reduce anxiety and the need for sedation. This approach can help centres with advanced scanners enhance their paediatric imaging services.

In addition to static imaging, fully dynamic PET studies combined with pharmacokinetic modelling can provide detailed physiological information on tracer kinetics, including delivery, metabolism, cellular transport function and receptor binding.^63^ Dynamic PET imaging with tracer kinetic modelling is more routinely employed in adult patients; parametric mapping in PET offers advantages like tumour-to-background contrast enhancement, providing quantitative, functional data on biological processes (e.g., blood flow, metabolism) that go beyond conventional static images, enabling earlier and more accurate assessment of disease and treatment response, improved diagnostic potential and a more robust understanding of a tracer’s pharmacokinetics and distribution over time and space^64^ Its feasibility, accuracy and clinical utility in paediatric populations remain to be systematically investigated, as the application in children requires short acquisition protocols to minimise motion artefacts and avoid sedation.

Finally, LAFOV PET seems to be cost-effective in the adult population compared with SAFOV PET. This may also be the case for the paediatric population, given the specific challenges faced and the limitations to high throughput due to time-intensive patient preparation, including anaesthesia.^65^

## Conclusion

LAFOV PET/CT offers a transformative approach to paediatric nuclear medicine. It enables significant reductions in radiation exposure and scan time, enhances patient comfort and supports a wide range of clinical applications—often without the need for sedation or anaesthesia. As access improves and protocols are standardised, LAFOV PET/CT is likely to play a key role in the future of clinical paediatric imaging.
